# Aesthetic-oriented breast reconstruction: a feasibility study of a novel technique combining Suture-Based Marking Scar Minimising and Axillary-Lateral Thoracic Fat Flap Reconstruction with Tissue Sliding (S-SMARTS)

**DOI:** 10.1007/s00595-026-03285-1

**Published:** 2026-05-23

**Authors:** Hui Ying Khoo, Yoshiaki Sota, Kenzo Shimazu

**Affiliations:** 1 Department of Breast and Endocrine Surgery, Graduate School of Medicine, Osaka University, Suita, Japan; 2 Breast and Endocrine Unit, Surgical Department, Hospital Sultan Ismail, Johor Bahru, Malaysia

**Keywords:** Hidden scar breast surgery, Breast-conserving surgery, S-SMARTS technique

## Abstract

Breast-conserving surgery with radiotherapy offers outcomes comparable to those of mastectomy. However, margin control and cosmetic results remain challenging, especially for deep or unfavorably located tumors. This study evaluated the S-SMARTS technique (suture-based marking, scar minimizing, and axillary-lateral thoracic fat flap reconstruction with tissue sliding), a hidden-scar, volume displacement method using axillary and lateral thoracic fat flaps. In this single-center retrospective study (Osaka University, Jan 2020–Apr 2025), 44 patients underwent breast-conserving surgery using S-SMARTS. The margin status and cosmetic outcomes (via BCCT.core) were analyzed. Negative margins were achieved in 95.5% of cases on the initial excision and 100% after intraoperative frozen section. The BCCT.core assessment demonstrated that 76.2% of the cases had “Good/Excellent” and 23.8% had “Fair” outcomes. Multivariate analyses were performed to explore any potential predictors of margin positivity. However, no statistically robust predictors were identified. S-SMARTS demonstrates technical feasibility and acceptable early oncologic and aesthetic outcomes in a single-surgeon cohort. Further comparative and multi-institutional studies are warranted to validate its oncologic impact, reproducibility, and patient-reported aesthetic benefits.

## Introduction

Breast-conserving surgery (BCS) is the preferred approach for early-stage breast cancer, offering oncologic safety comparable to that of mastectomy with superior breast preservation [1]. Techniques have evolved from conventional BCS to oncoplastic procedures, regional flaps, and minimally invasive methods, such as endoscopic and robot-assisted surgery. However, these often require specialized skills, equipment, and longer learning curves [2, 3].

Accurate tumor localization is critical, especially for non-palpable lesions. Wire-guided, radioactive, and magnetic seed methods exist but can be resource-intensive. Ultrasound-guided localization offers improved margin clearance and fewer reoperations; however, BCS still shows margin positivity rates of 6–20% [4, 5], and the aesthetic outcomes vary widely [6–9]. This underscores the need for simpler, effective alternatives.

The S-SMARTS (Suture-based Marking, Scar-Minimizing, and Axilla-lateral thoracic fat flap Reconstruction with Tissue Sliding) technique was developed to address these challenges. It integrates ultrasound-guided suture mapping, margin dye marking, and fat flap reconstruction via hidden incisions, thereby avoiding the use of complex tools. This study describes the S-SMARTS technique and evaluates its safety, complication rates, and cosmetic results as a reproducible, scar-sparing alternative to current BCS methods.

Unlike established oncoplastic volume displacement techniques that emphasize glandular rearrangement or formal regional flap reconstruction, S-SMARTS was designed as an integrated workflow centered on lateral access resection, combining four principles: (1) suture-based ultrasound-guided pre-resection marking to optimize margin-oriented excision while preserving scar orientation; (2) traction-assisted mobilization lateralizes medial or central tumors, permitting their delivery and excision through a small lateral incision despite restricted visualization; (3) selective use of axillary-lateral thoracic fat as a limited volume displacement source rather than a formal regional flap; and (4) tissue sliding to redistribute closure tension away from the excision cavity.

Notably, once resection is completed through the lateral/axillary field, principles (3) and (4) provide the most straightforward and anatomically compatible methods for contour restoration and tension redistribution from the same operative window. Collectively, these elements distinguish S-SMARTS from conventional oncoplastic techniques by redefining the incision strategy and embedding reconstruction within the lateral-access workflow.

The conceptual aim of S-SMARTS is not to replace level II oncoplastic procedures, but to provide a simplified, resource-conscious approach for challenging tumor locations where conventional breast-conserving surgery risks unfavorable scarring or contour deformity.

## Methodology

We retrospectively reviewed the data of 50 patients who underwent S-SMARTS breast-conserving surgery by a single surgeon (Jan 2021–Apr 2025). The inclusion criteria were early-stage breast cancer and suitability for S-SMARTS. We excluded cases with contralateral surgery, diagnostic excision only, or superficial tumors requiring skin removal. After exclusions, 44 patients were included in the analysis. This study was approved by the Institutional Ethics Committee of Osaka University (Approval No. K25093).

Preoperative MRI and ultrasound examinations were performed. Skin marking was done one day prior in a position simulating surgery. Under ultrasound guidance, lesions and their extensions were marked with a 1.5–2.0 cm margin. Symmetry was checked seated. The incisions were tailored to the location of the tumor: lateral for the outer/upper inner quadrants, peri-areolar or inframammary for central/lower inner tumors. Sub-axillary and lateral fat tissues were evaluated for reconstruction, and the lateral thoracic perforator was marked to preserve vascularity.

This retrospective technical feasibility analysis did not include a comparative control group.

### Surgical procedure

#### Removal of the target lesion

**Fig. 1:**
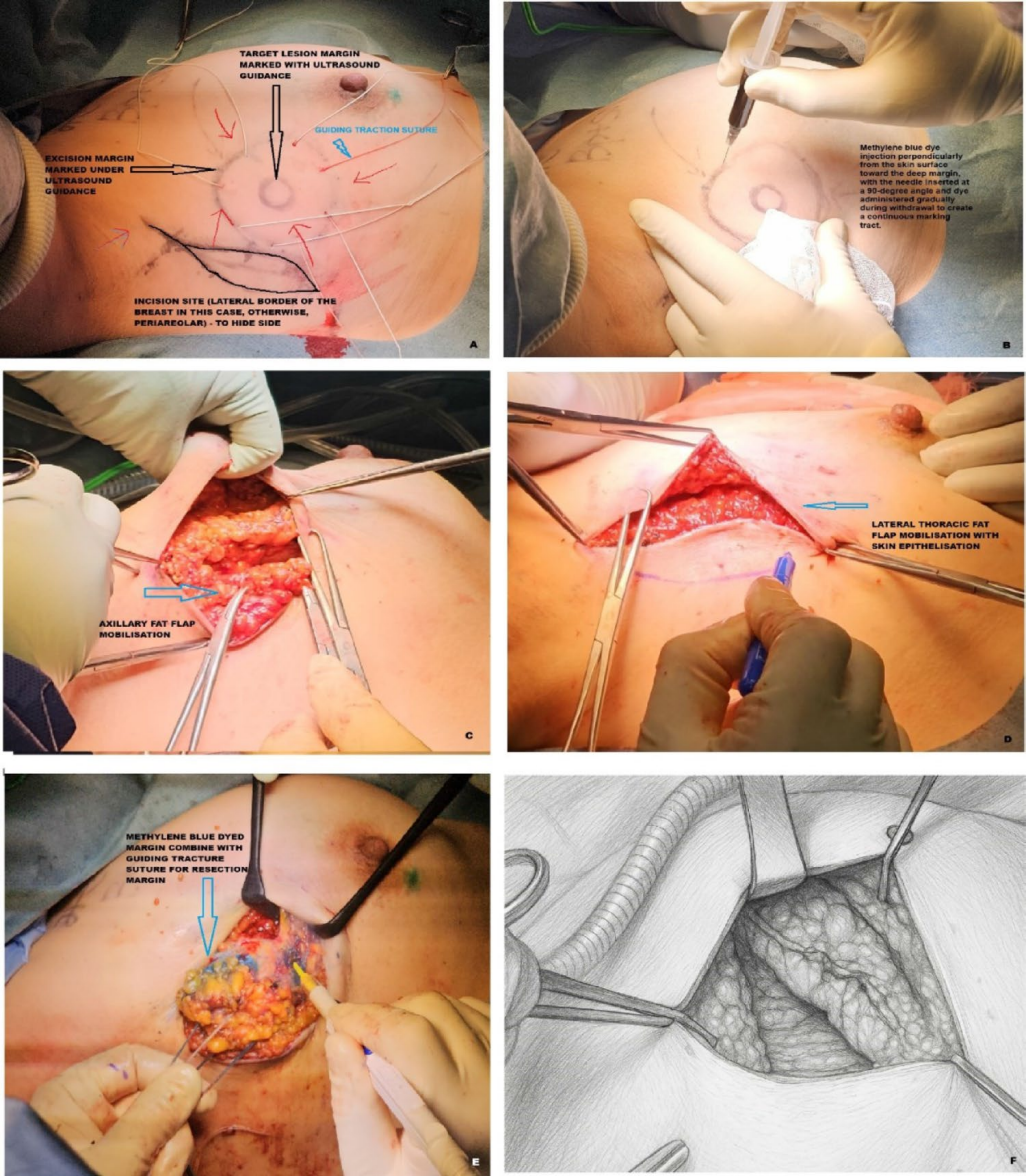
. Stepwise illustration of the S-SMARTS technique. (**A**) Preoperative surface marking of the target lesion and planned excision margin under ultrasound guidance, with placement of guiding traction sutures and incision site design. (**B**) Perpendicular injection of methylene blue from the skin surface toward the anticipated deep resection margin at a 90-degree angle, with gradual dye release during needle withdrawal to create a continuous marking tract. (**C**) Mobilisation of the axillary fat flap following tumour excision. (**D**) Mobilisation of the lateral thoracic fat flap with preservation of skin epithelialisation for defect reconstruction. (**E**) Traction-assisted lateralization of a medially located lesion. Guiding traction sutures elevate and mobilize the tumor toward the lateral incision, enhancing visualization of the methylene blue–marked deep margin and converting a visually restricted medial field into a controlled operative plane for precise excision. (**F**) Schematic illustration demonstrating the final resection cavity and flap arrangement

On the day of surgery, the lesion boundaries were confirmed using ultrasound (Fig. 1A). Blue dye was injected along the pre-marked margins (Fig. 1B), and directional silk sutures were placed circumferentially for traction and dissection (Fig. 1A). Dual-method SLNB was performed concurrently with tumor resection. Dissection followed a skin-sparing mastectomy approach, preserving the skin envelope. The lesions were excised along the dye-marked margins using sequential traction. The combined use of methylene blue margin delineation and deep directional traction sutures provides a three-dimensional resection guide by defining both the surface and depth of the excision plane (Fig. 1E). This facilitates accurate deep margin control and minimizes the skewing of the resection trajectory that may occur when relying solely on skin surface markings, particularly in deep cavities and unfavorable locations. Margin shaving, including the nipple base if indicated, was submitted for a frozen section analysis.

## Reconstruction

Volume displacement reconstruction utilized axillary and lateral thoracic fat to maintain the contour (Fig. 1C-D). The perforators were preserved to reduce fat necrosis. Redundant skin was denuded, and both breasts were draped for symmetry. The contour was reassessed in a semi-upright position before closure (Fig. 1F).

## Data collection and statistical analysis

Clinical, radiological, and pathological data were retrospectively retrieved from hospital records. Breast volume was measured using Synapse Vincent MRI software, and ptosis was graded using the Regnault classification. Cosmetic outcomes were assessed using the BCCT. core licensed software. A 20% tumor-to-breast volume threshold (per ASCO guidelines) defined high-volume excision. Positive margins were defined as tumor on ink (invasive) or < 2 mm (DCIS). Data were analyzed using R Studio and JMP. Given the limited number of outcome events, all regression analyses were considered exploratory and hypothesis-generating, and the results were interpreted with caution to avoid overfitting.

## Results

### Demographic characteristics

Of the 44 women (mean age, 55.5 ± 11.2 years; body mass index, 21.8 ± 3.4 kg/m²), most had small (< 400 cm³, 68.2%), round (51.2%), or flat (25.6%) breasts, and BI-RADS C density (61.4%). No ptosis was observed in 50%. The mean tumor size was 15.5 ± 9.7 mm, mainly in the upper-outer (50%) and upper-inner (34.1%) quadrants. Invasive ductal carcinoma (70.5%) and the luminal subtype (79.6%) were predominant. Neoadjuvant chemotherapy was given to 4.6%. SLNB was performed in 95.5%. The mean specimen weight was 43.3 g, and 81.8% had < 20% of the breast volume resected. One hematoma (2.3%) occurred; no other complications were noted (Table 1).

**Table 1:** Patient Demographics, Tumor Characteristics, and Outcomes (n = 44)

Section	Category	SD	N	%
Demographics	Age (years)	55.5 ± 11.2	44	100
	BMI (kg/m^2^)	21.8 ± 3.4	44	100
Baseline breast profile	Breast Volume (cm^3^)	356.6 ± 228.1	44	
	Breast Size: L (> 600 cm^3^)		6	13.6
	Breast Size: M (400–600 cm^3^)		8	18.2
	Breast Size: S (< 400 cm^3^)		30	68.2
	Shape: Asymmetric		1	2.3
	Shape: Flat		11	25.6
	Shape: Pendulous		9	20.9
	Shape: Round		22	51.2
	Density: B (Scattered)		14	31.8
	Density: C (Heterogeneously dense)		27	61.4
	Density: D (Extremely dense)		3	6.8
	Ptosis Grade: 0		29	65.9
	Ptosis Grade: 1		8	18.2
	Ptosis Grade: 2		5	11.4
	Ptosis Grade: 3		1	2.3
	Ptosis Grade: No imaging		1	2.3
	Resected Volume (g)	43.3 ± 26.9	44	
	Tumor:Breast volume ratio < 20%		36	81.8
	Tumor:Breast volume ratio ≥ 20%		8	18.2
Section	Category	SD	N	%
Tumor Characteristics	Tumor Size (mm)	15.5 ± 9.7	44	
	Location: A		15	34.1
	Location: B		1	2.3
	Location: C		22	50.0
	Location: D		5	11.4
	Location: E		1	2.3
	Multifocal: No		39	88.6
	Multifocal: Yes		5	11.4
	Pathology: DCIS		7	15.9
	Pathology: Invasive Ductal CA		31	70.5
	Pathology: Invasive Lobular CA		4	9.1
	Pathology: Other		2	4.6
	Biology: Her2 +		3	6.8
	Biology: Luminal		35	79.6
	Biology: Triple negative		3	6.8
	Biology: Triple positive		3	6.8
Surgical Details	Centripetal incision: No		37	84.1
	Centripetal incision: Yes		7	15.9
	Neoadjuvant Chemo: No		42	95.5
	Axillary dissection: Level I		2	4.6
	Axillary dissection: SLNB		42	95.5
	Reoperation needed: No		44	100
Outcomes	Initial margin negative		42	95.5
	Initial margin positive		2	4.5
	BCCT Score: Good/Excellent		32	76.2
	BCCT Score: Fair		10	23.8
	BCCT Score: No imaging		2	-

## Primary outcome: positive surgical margin and its associated factors

**Fig. 2:**
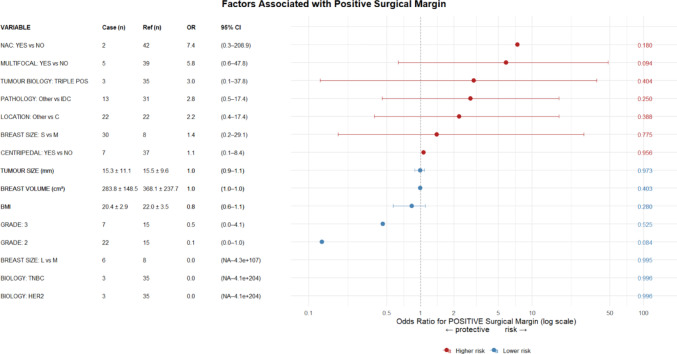
Factors associated with positive surgical margin

Initial negative margins were achieved in 95.4% (42/44) of cases (Table 1); the remaining two were cleared intraoperatively via a frozen section analysis, thus avoiding reoperation. The factors associated with positive margins included multifocality, neoadjuvant therapy, DCIS, ILC, and aggressive subtypes. However, none of these factors were found to be statistically significant (Fig. 2). The interpretation of the margin status must be contextualized by the routine use of intraoperative frozen section analysis, which limits the attribution of margin control solely to the surgical technique.

## Secondary outcome: predictors of aesthetic results based on the BCCT score

**Fig. 3:**
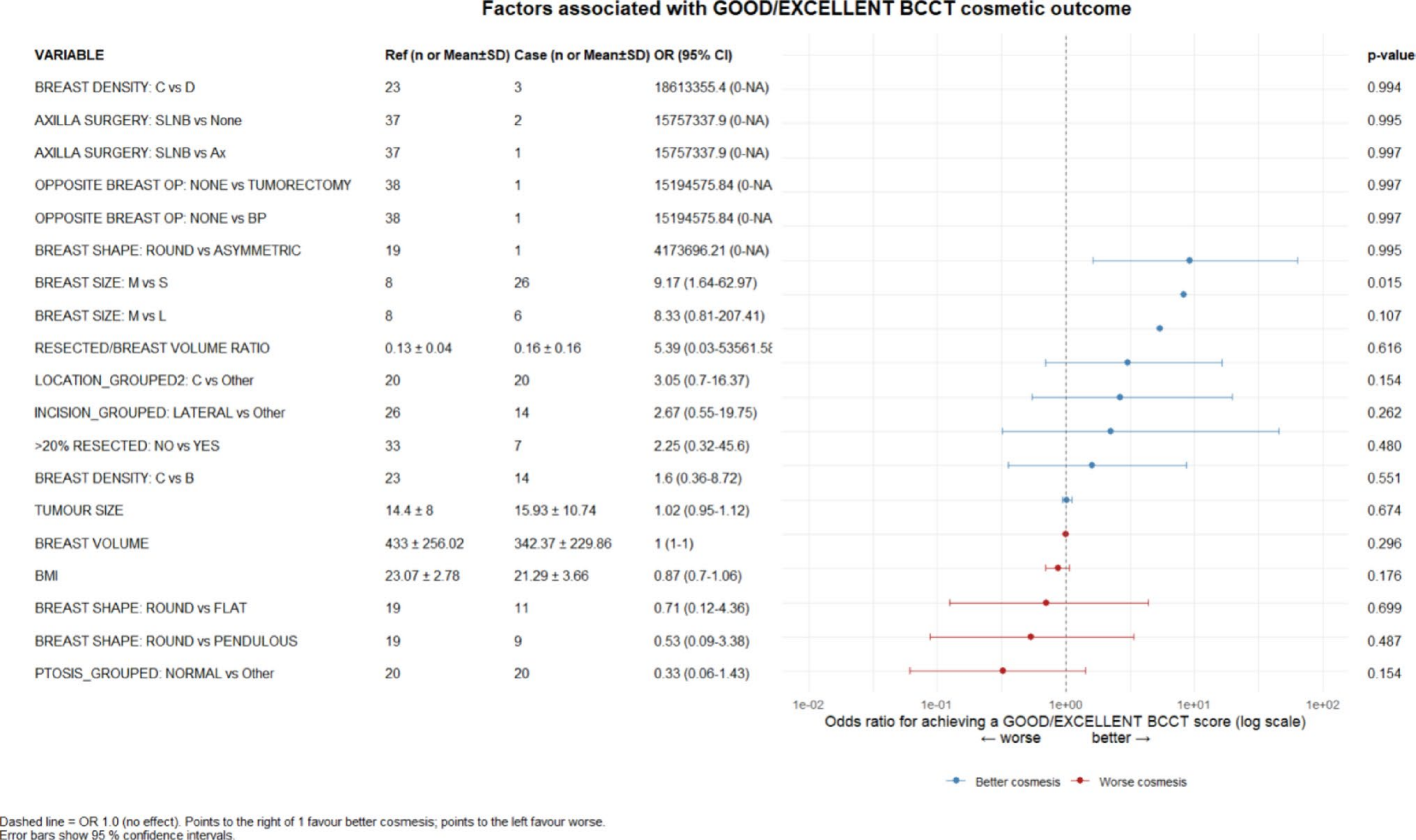
Factors associated with cosmetic outcome

BCCT.core rated outcomes as good or excellent in 76.2% and fair in 23.8% (**Table I**). Better cosmetic scores were associated with medium (OR: 9.17) and large (OR: 8.33) breasts and higher resection-to-volume ratios (OR: 5.39). Lower scores were associated with grade 1 ptosis and flat or pendulous shapes. The tumor site, incision, and contralateral surgery had no consistent effect (Fig. 3). These trends may aid aesthetic expectations in BCS. Odds ratios with wide confidence intervals should be interpreted as descriptive trends rather than definitive predictors.

## Discussion

A key contributor to margin control is the combined use of methylene blue marking and deep traction sutures, which helps maintain alignment between superficial and deep margins and reduces tangential excision, particularly in deep or medially located cavities. This technique enhances spatial orientation during resection and improves technical reproducibility for the operating surgeon. In this series, an initially negative resection margin was achieved in 42 of 44 cases without an intraoperative frozen section analysis, increasing to 44 of 44 cases with the use of a frozen section analysis. These results compare favorably with historical reports and suggest that the S-SMARTS technique provides a robust platform for achieving adequate margins [4, 5]. Nevertheless, the routine use of intraoperative frozen sections represents an important confounding factor. Accordingly, the low rate of positive margins observed in this single-surgeon study should be interpreted with caution, as margin outcomes reflect an integrated surgical-pathological strategy rather than the independent oncologic effect of the S-SMARTS technique alone. Furthermore, the reproducibility, learning curve, and inter-operator variability were not formally assessed and warrant evaluation in future multi-surgeon prospective studies [3].

Aesthetic results were favorable (76.2% good/excellent on BCCT.core), comparable to the reported outcomes of established oncoplastic techniques (60–90%) [2, 8, 9]. Although BCCT.core provides a standardized and objective assessment, it is recognized to underrepresent scar visibility and patient-perceived cosmetic satisfaction [10]. In addition, the lack of validated patient-reported outcome measures, such as BREAST-Q and blinded panel evaluation, represents a significant limitation [7, 11]. Therefore, the aesthetic outcomes in this study should be interpreted primarily as objective photographic assessments rather than comprehensive patient-centered evaluations. Future studies incorporating multimodal aesthetic and quality-of-life measures are required to more fully characterize the patient-reported benefits of this technique [6].

## Conclusion

S-SMARTS is a feasible scar-conscious breast-conserving surgical approach structured around lateral-access resection, integrating margin-oriented excision with limited volume displacement. In this single-center, single-surgeon retrospective series, the technique demonstrated acceptable early oncologic and aesthetic outcomes. However, conclusions regarding oncologic superiority, reproducibility, and cosmetic advantage are limited by the study design. Prospective comparative studies incorporating patient-reported outcomes are required before broader adoption can be recommended.
